# Bis(tetra­phenyl­phospho­nium) di-μ-iodido-bis[di­iodido­tellurate(II)]

**DOI:** 10.1107/S1600536808035927

**Published:** 2008-11-20

**Authors:** Sari M. Närhi, Raija Oilunkaniemi, Risto S. Laitinen

**Affiliations:** a Department of Chemistry, P.O. Box 3000, FI-90014 University of Oulu, Finland

## Abstract

The structure of the title compound, (C_24_H_20_P)_2_[Te_2_I_6_], is composed of discrete PPh_4_^+^ cations and centrosymmetric [Te_2_I_6_]^2−^ anions. The tellurium(II) atom shows a sligthly distorted square-planar TeI_4_ geometry and is coordinated to two bridging and two terminal iodine atoms. The planar [Te_2_I_6_]^2−^ ions are isolated by the cations and no inter­molecular tellurium–halogen or halogen–halogen inter­actions are present.

## Related literature

For a review of halidotellurate anions, see Krebs & Ahlers (1990 ▶). For the structure of the [Te_2_I_6_]^2−^ anion, see: Konu & Chivers (2006 ▶); Fujiwara *et al.* (2002 ▶). For related materials, see: Janickis *et al.* (2002 ▶, 2003 ▶).
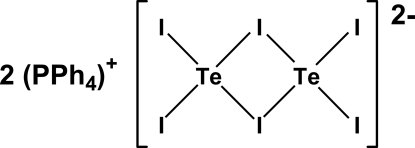


## Experimental

### 

#### Crystal data

(C_24_H_20_P)_2_[Te_2_I_6_]
*M* *_r_* = 1695.34
Monoclinic, 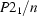

*a* = 13.252 (3) Å
*b* = 14.494 (3) Å
*c* = 14.109 (3) Å
β = 107.48 (3)°
*V* = 2584.8 (9) Å^3^
*Z* = 2
Mo *K*α radiation
μ = 4.80 mm^−1^
*T* = 100 (2) K
0.15 × 0.15 × 0.10 mm

#### Data collection

Bruker–Nonius KappaCCD diffractometer
Absorption correction: multi-scan (*SADABS*; Sheldrick, 1996 ▶) *T*_min_ = 0.511, *T*_max_ = 0.619
23569 measured reflections
5009 independent reflections
4225 reflections with *I* > 2σ(*I*)
*R*_int_ = 0.103

#### Refinement

*R*[*F* ^2^ > 2σ(*F* ^2^)] = 0.041
*wR*(*F* ^2^) = 0.107
*S* = 1.02
5009 reflections
263 parameters
H-atom parameters constrained
Δρ_max_ = 1.08 e Å^−3^
Δρ_min_ = −1.03 e Å^−3^

### 

Data collection: *COLLECT* (Nonius, 1998 ▶); cell refinement: *DENZO-SMN* (Otwinowski & Minor, 1997 ▶); data reduction: *DENZO-SMN*; program(s) used to solve structure: *SIR92* (Altomare *et al*., 1993 ▶); program(s) used to refine structure: *SHELXL97* (Sheldrick, 2008 ▶); molecular graphics: *DIAMOND* (Brandenburg & Berndt, 2008 ▶); software used to prepare material for publication: *WinGX* (Farrugia, 1999 ▶).

## Supplementary Material

Crystal structure: contains datablocks I, global. DOI: 10.1107/S1600536808035927/hb2834sup1.cif

Structure factors: contains datablocks I. DOI: 10.1107/S1600536808035927/hb2834Isup2.hkl

Additional supplementary materials:  crystallographic information; 3D view; checkCIF report

## Figures and Tables

**Table 1:** Selected bond lengths (Å)

Te1—I2	2.8103 (8)
Te1—I1	2.8590 (8)
Te1—I3	3.0676 (8)
Te1—I3^i^	3.2244 (8)

Symmetry code: (i) 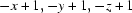.

**Fig. 1.**
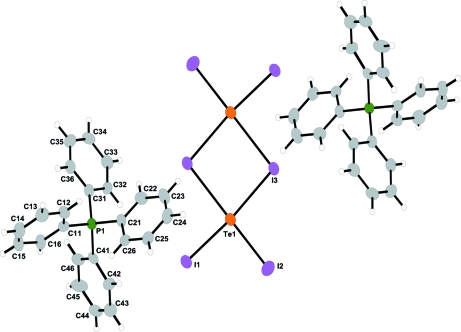
The molecular structure of (I) showing displacement ellipsoids drawn at 50% probability (arbitrary spheres for the H atoms). The unlabelled atoms are generated by the symmetry operation (1–x, 1–y, 1–z).

**Fig. 2.**
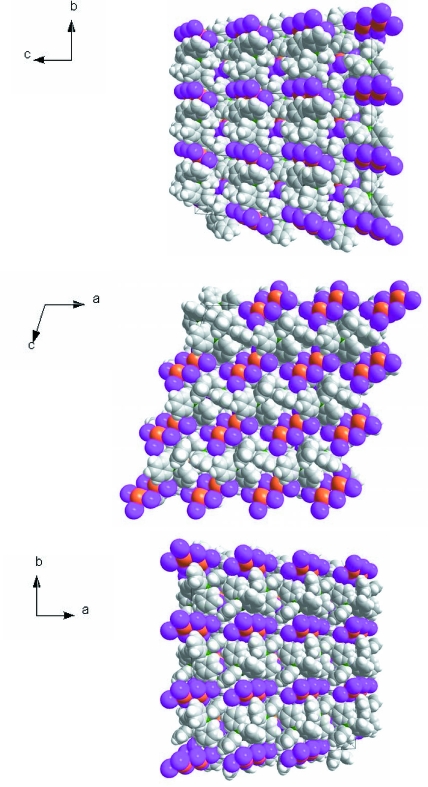
Space filling representations of the packing of the molecules.

## supplementary crystallographic information

### Comment

The asymmetric unit of the title compound, (I), [PPh_4_]_2_[Te_2_I_6_], consists of one tetraphenylphosphonium cation and half of the anion (Fig. 1). The tellurium atoms show a distorted square planar coordination geometry and are coordinated to two bridging and two terminal iodine atoms (Table 1). The terminal Te—I bond lengths of 2.8103 (8) Å and 2.8590 (8) Å as well as the bridging Te—I bond lengths of 3.0676 (8) Å and 3.2244 (8) Å can be compared to the corresponding Te—I bonds in [(Et_3_PO)_2_H]_2_[Te_2_I_6_] (Konu & Chivers, 2006) and (C_10_H_8_S_8_)_2_[Te_2_I_6_].3(C_10_H_8_S_8_) (Fujiwara *et al.* 2002).  In [(Et_3_PO)_2_H]_2_[Te_2_I_6_] and (C_10_H_8_S_8_)_2_[Te_2_I_6_].3(C_10_H_8_S_8_) the anions are involved in interionic I···I interactions shorter than the van der Waals radii of two iodine atoms, whereas in the present compound intermolecular iodine-iodine contacts are absent. The planar [Te_2_I_6_]^2-^ ions are isolated by the cations as shown in Fig. 2.

The present salt was obtained from the reaction mixture of PPh_4_Cl, KI, Te, TeI_4_, and I_2_ in acetonitrile. Corresponding reactions with selenium, tellurium and bromine containing starting materials have yielded interesting mixed-valence bromidotellurate(IV)-selenate(II) and -selenate(I) anions [for illustrative examples, see Janickis *et al.* (2002, 2003)].

### Experimental

The mixture of PPh_4_Cl (0.3750 g, 1.00 mmol), KI (0.2 g, 1 mmol), Te (0.1452 g, 1.14 mmol), TeI_4_ (0.3172 g, 0.50 mmol), and I_2_ (0.1274 g, 0.50 mmol) in 15 ml acetonitrile gave a grey precipitate and a dark red solution after refluxing 2 h. A mixture of crystals of (I) and PPh_4_I_3_ was isolated from the filtrate after subsequent concentration of the solution.

### Refinement

The H atoms were positioned geometrically (C—H = 0.95 Å) and refined as riding with *U*_iso_(H) = 1.2 *U*_eq_(C).

### Crystal data

**Table:**

(C_24_H_20_P)_2_[Te_2_I_6_]	*F*_000_ = 1560
*M**_r_* = 1695.34	*D*_x_ = 2.178 Mg m^−^^3^
Monoclinic, *P*2_1_/*n*	Mo *K*α radiation λ = 0.71073 Å
Hall symbol: -P 2yn	Cell parameters from 4225 reflections
*a* = 13.252 (3) Å	θ = 3.0–26.0º
*b* = 14.494 (3) Å	µ = 4.81 mm^−^^1^
*c* = 14.109 (3) Å	*T* = 100 (2) K
β = 107.48 (3)º	Plate, brown
*V* = 2584.8 (9) Å^3^	0.15 × 0.15 × 0.10 mm
*Z* = 2	

### Data collection

**Table:**

Bruker Nonius KappaCCD diffractometer	5009 independent reflections
Radiation source: fine-focus sealed tube	4225 reflections with *I* > 2σ(*I*)
Monochromator: graphite	*R*_int_ = 0.103
*T* = 100(2) K	θ_max_ = 26.0º
φ scans, and ω scans with κ offsets	θ_min_ = 3.0º
Absorption correction: multi-scan(SADABS; Sheldrick, 1996)	*h* = −16→16
*T*_min_ = 0.511, *T*_max_ = 0.619	*k* = −17→17
23569 measured reflections	*l* = −17→16

### Refinement

**Table:**

Refinement on *F*^2^	Hydrogen site location: inferred from neighbouring sites
Least-squares matrix: full	H-atom parameters constrained
*R*[*F*^2^ > 2σ(*F*^2^)] = 0.041	*w* = 1/[σ^2^(*F*_o_^2^) + (0.0555*P*)^2^ + 5.4856*P*] where *P* = (*F*_o_^2^ + 2*F*_c_^2^)/3
*wR*(*F*^2^) = 0.107	(Δ/σ)_max_ = 0.001
*S* = 1.03	Δρ_max_ = 1.09 e Å^−^^3^
5009 reflections	Δρ_min_ = −1.03 e Å^−^^3^
263 parameters	Extinction correction: SHELXL, Fc^*^=kFc[1+0.001xFc^2^λ^3^/sin(2θ)]^-1/4^
Primary atom site location: structure-invariant direct methods	Extinction coefficient: 0.00320 (19)
Secondary atom site location: difference Fourier map	

### Special details

**Table:**

Geometry. All e.s.d.'s (except the e.s.d. in the dihedral angle between two l.s. planes) are estimated using the full covariance matrix. The cell e.s.d.'s are taken into account individually in the estimation of e.s.d.'s in distances, angles and torsion angles; correlations between e.s.d.'s in cell parameters are only used when they are defined by crystal symmetry. An approximate (isotropic) treatment of cell e.s.d.'s is used for estimating e.s.d.'s involving l.s. planes.
Refinement. Refinement of *F*^2^ against ALL reflections. The weighted *R*-factor *wR* and goodness of fit *S* are based on *F*^2^, conventional *R*-factors *R* are based on *F*, with *F* set to zero for negative *F*^2^. The threshold expression of *F*^2^ > σ(*F*^2^) is used only for calculating *R*-factors(gt) *etc*. and is not relevant to the choice of reflections for refinement. *R*-factors based on *F*^2^ are statistically about twice as large as those based on *F*, and *R*- factors based on ALL data will be even larger.

### Fractional atomic coordinates and isotropic or equivalent isotropic displacement parameters (Å^2^)

**Table:**

	*x*	*y*	*z*	*U*_iso_*/*U*_eq_	
Te1	0.39145 (3)	0.49167 (2)	0.59757 (3)	0.02321 (13)	
I1	0.18308 (3)	0.56474 (3)	0.56278 (3)	0.02848 (14)	
I2	0.39684 (3)	0.41640 (3)	0.78259 (3)	0.03878 (15)	
I3	0.61278 (3)	0.41817 (3)	0.61252 (3)	0.02757 (13)	
P1	0.32106 (11)	0.11106 (9)	0.54406 (10)	0.0195 (3)	
C11	0.2712 (4)	−0.0042 (4)	0.5160 (4)	0.0229 (12)	
C12	0.3382 (5)	−0.0802 (4)	0.5366 (5)	0.0270 (13)	
H12	0.4121	−0.0718	0.5661	0.032*	
C13	0.2973 (5)	−0.1679 (4)	0.5144 (4)	0.0316 (13)	
H13	0.3432	−0.2198	0.5295	0.038*	
C14	0.1894 (5)	−0.1807 (4)	0.4700 (4)	0.0330 (14)	
H14	0.1613	−0.2411	0.4555	0.040*	
C15	0.1229 (5)	−0.1046 (4)	0.4470 (5)	0.0304 (13)	
H15	0.0496	−0.1133	0.4146	0.036*	
C16	0.1620 (5)	−0.0163 (4)	0.4706 (4)	0.0268 (12)	
H16	0.1157	0.0353	0.4564	0.032*	
C21	0.4391 (4)	0.1058 (4)	0.6475 (4)	0.0216 (11)	
C22	0.5306 (4)	0.1552 (4)	0.6471 (4)	0.0268 (12)	
H22	0.5306	0.1916	0.5911	0.032*	
C23	0.6208 (5)	0.1507 (4)	0.7286 (5)	0.0323 (14)	
H23	0.6825	0.1841	0.7285	0.039*	
C24	0.6208 (5)	0.0973 (4)	0.8100 (4)	0.0305 (13)	
H24	0.6829	0.0934	0.8652	0.037*	
C25	0.5306 (5)	0.0497 (4)	0.8112 (4)	0.0263 (12)	
H25	0.5307	0.0144	0.8680	0.032*	
C26	0.4407 (4)	0.0530 (4)	0.7304 (4)	0.0223 (11)	
H26	0.3796	0.0191	0.7314	0.027*	
C31	0.3505 (4)	0.1619 (4)	0.4396 (4)	0.0208 (11)	
C32	0.3740 (4)	0.2572 (4)	0.4442 (4)	0.0233 (11)	
H32	0.3701	0.2926	0.4996	0.028*	
C33	0.4029 (4)	0.2990 (4)	0.3680 (4)	0.0268 (12)	
H33	0.4193	0.3630	0.3711	0.032*	
C34	0.4077 (4)	0.2469 (4)	0.2870 (4)	0.0266 (12)	
H34	0.4288	0.2752	0.2353	0.032*	
C35	0.3818 (4)	0.1534 (4)	0.2808 (4)	0.0267 (12)	
H35	0.3834	0.1190	0.2240	0.032*	
C36	0.3537 (4)	0.1101 (4)	0.3574 (4)	0.0247 (12)	
H36	0.3370	0.0462	0.3536	0.030*	
C41	0.2233 (4)	0.1829 (4)	0.5724 (4)	0.0221 (11)	
C42	0.2246 (5)	0.1973 (4)	0.6707 (4)	0.0301 (13)	
H42	0.2766	0.1680	0.7235	0.036*	
C43	0.1496 (5)	0.2545 (4)	0.6908 (5)	0.0357 (15)	
H43	0.1511	0.2652	0.7577	0.043*	
C44	0.0728 (5)	0.2959 (4)	0.6140 (5)	0.0314 (14)	
H44	0.0219	0.3353	0.6281	0.038*	
C45	0.0702 (5)	0.2800 (4)	0.5171 (5)	0.0342 (14)	
H45	0.0164	0.3078	0.4645	0.041*	
C46	0.1445 (5)	0.2242 (4)	0.4954 (5)	0.0296 (13)	
H46	0.1421	0.2139	0.4282	0.036*	

### Atomic displacement parameters (Å^2^)

**Table:**

	*U*^11^	*U*^22^	*U*^33^	*U*^12^	*U*^13^	*U*^23^
Te1	0.0210 (2)	0.0221 (2)	0.0260 (2)	−0.00164 (14)	0.00639 (16)	−0.00285 (14)
I1	0.0232 (2)	0.0348 (2)	0.0277 (2)	0.00469 (15)	0.00807 (16)	−0.00166 (15)
I2	0.0337 (3)	0.0505 (3)	0.0295 (3)	−0.00871 (18)	0.00551 (19)	0.00788 (18)
I3	0.0228 (2)	0.0278 (2)	0.0317 (2)	0.00296 (14)	0.00762 (17)	0.00327 (15)
P1	0.0181 (7)	0.0196 (7)	0.0205 (7)	0.0006 (5)	0.0053 (5)	−0.0005 (5)
C11	0.026 (3)	0.027 (3)	0.018 (3)	−0.002 (2)	0.010 (2)	0.002 (2)
C12	0.020 (3)	0.029 (3)	0.032 (3)	0.002 (2)	0.008 (3)	−0.003 (2)
C13	0.033 (3)	0.033 (3)	0.028 (3)	0.007 (3)	0.008 (3)	0.004 (3)
C14	0.044 (4)	0.025 (3)	0.029 (3)	−0.009 (3)	0.010 (3)	0.000 (3)
C15	0.026 (3)	0.031 (3)	0.030 (3)	−0.004 (2)	0.003 (2)	−0.004 (3)
C16	0.026 (3)	0.022 (3)	0.029 (3)	−0.002 (2)	0.004 (2)	−0.002 (2)
C21	0.018 (3)	0.020 (3)	0.026 (3)	0.001 (2)	0.004 (2)	−0.003 (2)
C22	0.027 (3)	0.024 (3)	0.031 (3)	0.006 (2)	0.011 (2)	0.003 (2)
C23	0.024 (3)	0.035 (3)	0.036 (3)	−0.006 (2)	0.005 (3)	0.001 (3)
C24	0.026 (3)	0.035 (3)	0.024 (3)	0.007 (2)	−0.003 (2)	−0.003 (2)
C25	0.026 (3)	0.027 (3)	0.024 (3)	0.006 (2)	0.005 (2)	−0.002 (2)
C26	0.022 (3)	0.024 (3)	0.021 (3)	0.004 (2)	0.007 (2)	−0.003 (2)
C31	0.018 (3)	0.023 (3)	0.019 (3)	0.001 (2)	0.002 (2)	0.002 (2)
C32	0.024 (3)	0.022 (3)	0.023 (3)	−0.001 (2)	0.007 (2)	−0.004 (2)
C33	0.024 (3)	0.024 (3)	0.032 (3)	0.002 (2)	0.008 (2)	0.005 (2)
C34	0.021 (3)	0.035 (3)	0.026 (3)	0.004 (2)	0.009 (2)	0.011 (2)
C35	0.029 (3)	0.027 (3)	0.025 (3)	0.005 (2)	0.008 (2)	−0.002 (2)
C36	0.023 (3)	0.020 (3)	0.029 (3)	0.000 (2)	0.005 (2)	−0.002 (2)
C41	0.015 (3)	0.023 (3)	0.028 (3)	−0.001 (2)	0.007 (2)	0.001 (2)
C42	0.023 (3)	0.046 (4)	0.021 (3)	0.006 (3)	0.006 (2)	−0.002 (3)
C43	0.030 (3)	0.037 (4)	0.043 (4)	0.000 (3)	0.016 (3)	−0.009 (3)
C44	0.026 (3)	0.028 (3)	0.046 (4)	0.001 (2)	0.020 (3)	−0.003 (3)
C45	0.036 (3)	0.030 (3)	0.046 (4)	0.013 (3)	0.024 (3)	0.016 (3)
C46	0.026 (3)	0.037 (3)	0.029 (3)	0.000 (2)	0.013 (3)	0.004 (3)

### Geometric parameters (Å, °)

**Table:**

Te1—I2	2.8103 (8)	C24—H24	0.9500
Te1—I1	2.8590 (8)	C25—C26	1.380 (8)
Te1—I3	3.0676 (8)	C25—H25	0.9500
Te1—I3^i^	3.2244 (8)	C26—H26	0.9500
I3—Te1^i^	3.2244 (8)	C31—C36	1.393 (8)
P1—C31	1.792 (6)	C31—C32	1.414 (7)
P1—C21	1.793 (6)	C32—C33	1.385 (8)
P1—C11	1.796 (6)	C32—H32	0.9500
P1—C41	1.799 (5)	C33—C34	1.386 (8)
C11—C12	1.389 (8)	C33—H33	0.9500
C11—C16	1.407 (8)	C34—C35	1.395 (8)
C12—C13	1.381 (8)	C34—H34	0.9500
C12—H12	0.9500	C35—C36	1.394 (8)
C13—C14	1.391 (9)	C35—H35	0.9500
C13—H13	0.9500	C36—H36	0.9500
C14—C15	1.387 (9)	C41—C46	1.396 (8)
C14—H14	0.9500	C41—C42	1.397 (8)
C15—C16	1.383 (8)	C42—C43	1.387 (8)
C15—H15	0.9500	C42—H42	0.9500
C16—H16	0.9500	C43—C44	1.381 (9)
C21—C26	1.393 (8)	C43—H43	0.9500
C21—C22	1.410 (8)	C44—C45	1.377 (9)
C22—C23	1.390 (8)	C44—H44	0.9500
C22—H22	0.9500	C45—C46	1.378 (8)
C23—C24	1.383 (9)	C45—H45	0.9500
C23—H23	0.9500	C46—H46	0.9500
C24—C25	1.385 (9)		
			
I2—Te1—I1	93.27 (3)	C26—C25—C24	120.5 (6)
I2—Te1—I3	92.54 (3)	C26—C25—H25	119.8
I1—Te1—I3	174.091 (17)	C24—C25—H25	119.8
I2—Te1—I3^i^	178.851 (17)	C25—C26—C21	120.2 (5)
I1—Te1—I3^i^	86.73 (3)	C25—C26—H26	119.9
I3—Te1—I3^i^	87.49 (3)	C21—C26—H26	119.9
Te1—I3—Te1^i^	92.51 (3)	C36—C31—C32	120.2 (5)
C31—P1—C21	109.5 (3)	C36—C31—P1	122.1 (4)
C31—P1—C11	110.9 (2)	C32—C31—P1	117.6 (4)
C21—P1—C11	108.2 (3)	C33—C32—C31	120.0 (5)
C31—P1—C41	107.1 (3)	C33—C32—H32	120.0
C21—P1—C41	110.8 (3)	C31—C32—H32	120.0
C11—P1—C41	110.2 (3)	C32—C33—C34	119.6 (5)
C12—C11—C16	120.1 (5)	C32—C33—H33	120.2
C12—C11—P1	121.5 (4)	C34—C33—H33	120.2
C16—C11—P1	118.5 (4)	C33—C34—C35	120.6 (5)
C13—C12—C11	120.1 (6)	C33—C34—H34	119.7
C13—C12—H12	120.0	C35—C34—H34	119.7
C11—C12—H12	120.0	C36—C35—C34	120.5 (5)
C12—C13—C14	120.3 (6)	C36—C35—H35	119.8
C12—C13—H13	119.9	C34—C35—H35	119.8
C14—C13—H13	119.9	C31—C36—C35	119.0 (5)
C15—C14—C13	119.7 (6)	C31—C36—H36	120.5
C15—C14—H14	120.2	C35—C36—H36	120.5
C13—C14—H14	120.2	C46—C41—C42	119.5 (5)
C16—C15—C14	120.9 (6)	C46—C41—P1	119.7 (4)
C16—C15—H15	119.6	C42—C41—P1	120.8 (4)
C14—C15—H15	119.6	C43—C42—C41	119.7 (6)
C15—C16—C11	119.0 (5)	C43—C42—H42	120.1
C15—C16—H16	120.5	C41—C42—H42	120.1
C11—C16—H16	120.5	C44—C43—C42	120.2 (6)
C26—C21—C22	119.2 (5)	C44—C43—H43	119.9
C26—C21—P1	119.7 (4)	C42—C43—H43	119.9
C22—C21—P1	121.1 (4)	C45—C44—C43	120.0 (6)
C23—C22—C21	119.9 (5)	C45—C44—H44	120.0
C23—C22—H22	120.0	C43—C44—H44	120.0
C21—C22—H22	120.0	C44—C45—C46	120.8 (6)
C24—C23—C22	119.9 (6)	C44—C45—H45	119.6
C24—C23—H23	120.1	C46—C45—H45	119.6
C22—C23—H23	120.1	C45—C46—C41	119.7 (6)
C23—C24—C25	120.3 (5)	C45—C46—H46	120.1
C23—C24—H24	119.9	C41—C46—H46	120.1
C25—C24—H24	119.9		
			
I2—Te1—I3—Te1^i^	−178.850 (17)	P1—C21—C26—C25	−178.9 (4)
I3^i^—Te1—I3—Te1^i^	0.0	C21—P1—C31—C36	107.5 (5)
C31—P1—C11—C12	92.1 (5)	C11—P1—C31—C36	−12.0 (5)
C21—P1—C11—C12	−28.1 (6)	C41—P1—C31—C36	−132.3 (4)
C41—P1—C11—C12	−149.4 (5)	C21—P1—C31—C32	−70.6 (5)
C31—P1—C11—C16	−86.9 (5)	C11—P1—C31—C32	169.9 (4)
C21—P1—C11—C16	152.9 (4)	C41—P1—C31—C32	49.6 (5)
C41—P1—C11—C16	31.6 (5)	C36—C31—C32—C33	−1.6 (8)
C16—C11—C12—C13	−1.2 (9)	P1—C31—C32—C33	176.6 (4)
P1—C11—C12—C13	179.8 (5)	C31—C32—C33—C34	0.5 (8)
C11—C12—C13—C14	0.9 (9)	C32—C33—C34—C35	1.2 (8)
C12—C13—C14—C15	0.7 (9)	C33—C34—C35—C36	−1.9 (8)
C13—C14—C15—C16	−2.1 (9)	C32—C31—C36—C35	0.9 (8)
C14—C15—C16—C11	1.8 (9)	P1—C31—C36—C35	−177.1 (4)
C12—C11—C16—C15	−0.2 (9)	C34—C35—C36—C31	0.8 (8)
P1—C11—C16—C15	178.9 (5)	C31—P1—C41—C46	36.7 (5)
C31—P1—C21—C26	−167.4 (4)	C21—P1—C41—C46	156.1 (4)
C11—P1—C21—C26	−46.3 (5)	C11—P1—C41—C46	−84.1 (5)
C41—P1—C21—C26	74.7 (5)	C31—P1—C41—C42	−143.9 (5)
C31—P1—C21—C22	13.4 (5)	C21—P1—C41—C42	−24.5 (5)
C11—P1—C21—C22	134.5 (4)	C11—P1—C41—C42	95.3 (5)
C41—P1—C21—C22	−104.6 (5)	C46—C41—C42—C43	−1.9 (9)
C26—C21—C22—C23	0.2 (8)	P1—C41—C42—C43	178.7 (5)
P1—C21—C22—C23	179.4 (5)	C41—C42—C43—C44	1.1 (9)
C21—C22—C23—C24	0.2 (9)	C42—C43—C44—C45	0.4 (9)
C22—C23—C24—C25	−1.1 (9)	C43—C44—C45—C46	−1.1 (9)
C23—C24—C25—C26	1.6 (9)	C44—C45—C46—C41	0.3 (9)
C24—C25—C26—C21	−1.2 (8)	C42—C41—C46—C45	1.2 (9)
C22—C21—C26—C25	0.3 (8)	P1—C41—C46—C45	−179.4 (5)

Symmetry codes: (i) −*x*+1, −*y*+1, −*z*+1.
